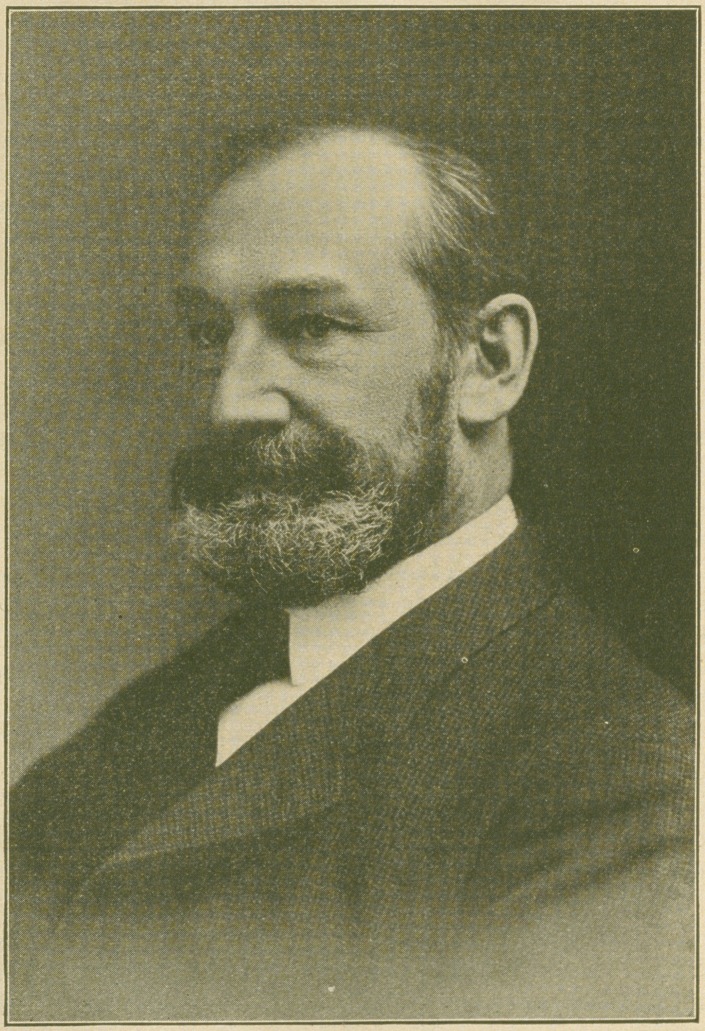# M. H. Fletcher, M. Sc., D. D. S., M. D.

**Published:** 1914-05-15

**Authors:** 


### OBITUARY.

M. H. Fletcher, M. Sc., D. D. S., M. D. Mordecia Hiatt Fletcher was born in Richmond, Indiana, September 18th, 1849, and died March 26th, 1914. His early life was spent in and near Richmond. He attended the country district school in the winter up to the age of 14 years. He was then apprenticed to a jeweler in Richmond for four years and later bought out the jeweler. About this time he also was a student in Earlham College. From 1870-77 he lived in New York and St. Louis, when he returned to

Richmond and began the study of Dentistry under Dr. J. W. Jay of Richmond. In the autumn of 1879 he entered the Ohio Dental College, Cincinnati, Ohio, graduating March, 1880.

He graduated from the Miami Medical College in 1884, having done all his Medical College work while practicing dentistry.

His research work began soon after completing his medical course; it has been largely in Biology, with special reference to the Pathology of the oral cavity and teeth. He has published no books, but has been a frequent contributor to dental and medical societies and journals, mostly reports of his own original researches in Physiology and Pathology. He served for six years as a member of the Ohio State Board of Dental Examiners, Anatomy Histology and Pathology being his subjects.

In 1887 by request he gave a paper before the Oral Surgery Section of the 9th International Medical Congress held at Washington, D. C., on "New Growths of the Pulp Chambers of Teeth of Mamaliae." This paper was the result of seven years of careful investigation.

In 1893 he was made chairman of the Section on Oral Surgery of the Pan-American Medical Congress. For fifteen years he served in some official capacity, as committeeman or officer in the Section on Stomatology of the American Medical Association. He has contributed a paper to that Society almost every year for the last twenty years. Among his valuable papers was one showing the untenable position of the process of Implantation. A paper describing these experiments was written for the Tenth International Medical Congress held in Berlin, 1890.

More recently his investigations have been along the lines of diseases of the mouth and teeth and their connection with the accessory air cavities of the nose and face and their bearing upon the general health. In July, 1895, he finished the course and received an academic degree in Electrical Science and Electro-Therapeutics in the National School of Electricity.

For more than twenty years he was closely connected with the management of the Cincinnati Society of Natural

History. Most of this time he was a member of its board of managers serving five times as president.

Probably because of his natural love for mechanics and because of his early training as a jeweler he had a strong propensity for inventions, and occasionally gave up his time and attention to these matters. He at one time invented a process for casting iron pipe for water mains that promised large financial gains, but for some cause unfortunately he never profited financially from this invention. He invented a number of dental appliances and processes for the more thorough accomplishment of his work. A set of bone drills and curettes have met with considerable approval by the profession interested in the treatment of peridental diseases involving the alveolar process.

In 1891 he took a course in Embryology at Earlham College and gave a course to the students at the same time in Photo-micrography.

In 1903 Earlham College bestowed on him the honorary degree of "Master of Science" for the work he had done along the lines of Scientific research. He took a course in Bacteriology at Ann Arbor under Professor F. G. Novy.

On Thursday March 26, 1914, about 10:30 A.M., while attending the needs of a patient Dr. Fletcher suffered a *hemorrhage of the brain* causing paralysis of the right face, arm and leg. He was conscious for nearly an hour and able to recognize his wife and daughter; also the physicians in attendance Drs. F. B. Samson, E. W. Mitchell, and F. W. Langdon. In about an hour he lapsed into unconsciousness, from which he did not rally. His death occurred about 4:30 P.M. of the same day (March 26, 1914.) There was at no time any evidence of pain or distress of any kind. The end came quietly and without struggle.

The sudden and untimely death of Dr. Fletcher takes from the professions one of its most efficient and valued workers in oral science. Like most of our scientific workers he carried his research work along with a heavy and exacting practice, and no doubt this double burden had its influence in breaking down his naturally rugged constitution. He was so thoroughly interested in the development of the better side of his beloved profession that he could not allow any time to be idly spent which could be put into scientific study or research. He was a constant attendant of dental society meetings and was interested in every feature of the program. He was able to discuss any paper whether on a technical or scientific subject, and because of his fair mindedness and respect for the opinions of others all essayists were glad when Fletcher gave their papers consideration. He was unselfish and never dogmatic, and yet it required a positive demonstration to satisfy his convictions Personally he was a perfect gentleman, always friendly and had a keen sense of the humorous which made him an admirable companion. He had hosts of friends and we never heard of an enemy, in fact, we don't recall ever hearing any one speak unkindly of him. Certainly that is a record for one to leave behind him, especially when the opportunities were so prevalent. We are inclined to think that any one who does things worth while in this world, is bound to make some enemies. Yet here was a man who had a most complex and strenuous career with all sorts and conditions of men, of whom every one will have some good word to say, and each will experience the loss of a friend when he learns of his death. We can not express our personal loss, it is too sudden and severe, it seems untrue. We rejoice that his life with all its strength and good cheer ran current with ours and that its influences have and will continue to bless and urge us on to greater and better efforts to attain those qualities of heart and mind which have made him the friend of all mankind as well as of his associates.

The sincere sympathy of the entire profession will be cordially extended to his beloved wife and daughter in their time of greatest sorrow.

## Figures and Tables

**Figure f1:**